# Peter Lee answers questions about additive manufacturing

**DOI:** 10.1038/s41467-020-17732-1

**Published:** 2020-08-17

## Abstract

Professor Peter D Lee is a materials scientist at the University College London. His group focuses on X-ray imaging and computational simulation of materials at a microstructural level for materials design and advanced manufacturing.

Tell us a little bit about you and what sparked your interest in additive manufacturing.

I came into additive manufacturing from the biomaterials side. About twenty-five years ago I worked with Larry Hench, the inventor of a bioactive material (specifically bioactive glass). He wanted to structure it to allow bone ingrowth. We had the idea of looking into additive manufacturing because it has the benefit of complete design freedom. At the same time, I was approached by the University of Liverpool in tandem with a private biomaterials company that wanted to modify their fabrication processes: they were looking at additive manufacturing to provide joint replacements with open cell surface structures for mechanical bone interlocking, and wanted to achieve more complex and larger interfaces—essentially lattices on their implants. That’s what really took off! For these projects, I used my background in modelling and in situ X-ray imaging for metrology and optimisation of component made by what was then a new additive manufacturing process—laser powder bed fusion.

While I have been doing modelling and imaging of flow in metals as they solidify for thirty-five years, I have always been aware of additive manufacturing because, at its core, it is a welding process: metal from a wire or a powder is added into a base plate, which is the definition of welding. What additive manufacturing brings is essentially whole new scientific challenges to welding. While welding has always been a fast process, the heat transfer rates in additive manufacturing are orders of magnitude faster, and four or five orders of magnitude faster than casting processes. It is a highly non-equilibrium process, which requires all new scientific insights. That was the challenge I saw, and that’s what piqued my interested. Unfortunately, 15 years ago synchrotrons weren’t readily available so we had to settle for doing ex situ imaging or relatively slow in situ imaging using laboratory sources.

How has additive manufacturing changed your field? Is there anything specific that it has enabled or made possible? Tell us how it impacted your research.

The largest impact of additive manufacturing has been to push the boundaries of spatial and temporal resolutions of in situ X-ray radiography. Traditional metal manufacturing is on the timescale of seconds, so you need 10−100 Hz to capture the phenomena. In additive manufacturing, you melt and solidify the weld pool in less than ten milliseconds, so you need to be running a hundred to a thousand times faster than this to capture the phenomena. That’s ten to a hundred thousand frames per second. Being able to achieve this has enabled us to see inside the weld pool during additive manufacturing to obtain all new insights, and these techniques and insights have been translated to many other phenomena in solidification and alloy design.

The other way it has impacted the field is in the design freedom: for polymers and biomaterials, the unparalleled design freedom enabling topological optimisation (or whole new designs) is almost the most important aspect. For metals, additive manufacturing is being developed first for high-value-added applications, such as aerospace and biomedical applications where the value added through designs that more energy efficient or performance enhancing are greatest. The caveat is that in both of these fields, components need to have an incredibly low failure rate and a long life. One of the weak points of metal additive manufacturing is the microstructure: because it is cooled so rapidly, it often has a higher strength but lower fatigue performance. And since in metals, many of the value-added applications are components that undergo cyclic fatigue, it is crucial we can understand, predict and optimise the microstructure.

What does additive manufacturing have to do to be more widely adopted? Are there any specific hurdles it has to overcome?

Additive manufacturing is really a process that is still in its infancy, and lessons learned from different approaches (e.g. printing for design freedom in polymers vs printing for mechanical and functional properties in metals) will feed into each other.

One key hurdle that isn’t well-researched is cost. There are many aspects to this, but two of the key ones are: (1) additive manufacturing machines are advancing so quickly that industries fabricating large number of components do not know how long they should amortise the cost of the machines for, and (2) the current machines are not energy efficient (when producing a large number of components, energy costs can be one of the largest cost components). This is because electricity is converted to laser light, which is then used to heat up powder material that may have a very low absorptivity. You then create spatter, ejecting away the powder feedstock you’re heating rather than forming the component.

Another challenge is understanding how to reuse the feedstock material: is it possible to reuse it multiple times? How do we recycle it? One of the many materials challenges is to determine how the surface changes to reused powder due to heating and oxidation affect its reuse. Using ultrafast synchrotron imaging we’re now quantifying this in situ and operando, so that you know how many times you may safely reuse it, rather than recycling.

For aerospace or high-value-added markets, where additive manufacturing is now used commercially, we still need to understand the microstructural features that limit life, and have accurate models that can predict these microstructures and the their impact on component lifing. We also need a better understanding of in situ heat treatments (which can help homogenise or modify the microstructure), so that we can use this thermal cycling to improve strength and ductility. As a simple example, the micro-segregation scale in cast and wrought alloys is ten of microns between the centre of the dendrite and the segregated interdendritic region, while in additive manufacturing it is on the order of a couple of microns—and the time required for diffusion to homogenise the composition goes with the square of the distance. This means that you may only need a hundredth of the heat treatment time to produce chemical equilibrium in additively manufactured structures.

Looking forward: where do you see additive manufacturing going next?

Additive manufacturing over the next five years… To me, additive manufacturing will remain an expensive process. It has to go into areas where it will inherently add tremendous value, such as applications with geometrical complexity that can’t be produced by other methods. However, we have to be able to predict the properties and lifing of these high-value components. But I think we can do this more accurately in additive manufacturing than other process—we are building in layers that are tens of microns thick, we can observe and record the microstructural features at this level as we fabricate a component. We could create a ‘digital twin’ that embeds a specific type of microstructural features and predict the resulting properties, updating our predictions depending on service conditions.

Today, when a component is manufactured via a cast and wrought process, both the alloy and the process are qualified using the average behaviour and a statistical distribution. This leads to large safety factors. Instead, we could envisage a component-by-component qualification. For example, if you make two components and measure as building one has features that give a predicted lifing of 2 months and one 20 years, you could direct the one for an emergency short-term application where you don’t need it to pass the fatigue life requirements of a 20-year application. What if you could more rapidly or cheaply source this particular part as a way to tie you over until your next full servicing? Additive manufacturing allows us to actually consider these types of fundamental questions and could enable us to transform the way we are doing things.

We are trying to develop process monitoring techniques informed and calibrated using synchrotron imaging, where we observe the features with the synchrotron and correlating them to low cost monitoring measurements (such as infrared and optical cameras). Using these we hope to create ‘digital twins’ which store the features and service conditions, adding real value to additive manufacturing, and justifying the extra cost.

Going forward, a lot of what we do in my group is ultrafast synchrotron imaging, and we are still having difficulties seeing microstructures because the X-ray attenuation between phases is small in many industrial alloys. However, fourth-generation synchrotron sources (such as the recently completed European Synchrotron Research Facility’s Extremely Brilliant Source, and similar upgrades to the Advanced Photon Source and the Diamond Light Source in the next few years) can offer increased coherency and the opportunity to better see the difference between solid, liquid and the many microstructural features we hope to resolve.

For you, is there a difference between additive manufacturing and 3D printing? If they are not equivalent, how do they differ?

There is no difference for me. I use the term 3D printing when speaking to the general public because they immediately understand and associate the idea to the manufacturing process. For research I use additive manufacturing: we need to better understand the fundamentals and the science, so the term additive manufacturing, which points to adding value, seems more appropriate.

3D printing is much more prevalent for polymers, which are much more consumer integrated. Alloys are more limited in their applications, more specialist and with higher value-added, which is probably why this field has stuck to the term ‘additive manufacturing’.

